# miR-5591-5p regulates the effect of ADSCs in repairing diabetic wound via targeting AGEs/AGER/JNK signaling axis

**DOI:** 10.1038/s41419-018-0615-9

**Published:** 2018-05-11

**Authors:** Qiang Li, Sizhan Xia, Yating Yin, Yanping Guo, Feifei Chen, Peisheng Jin

**Affiliations:** 1 Department of Plastic Surgery, Affiliated Hospital of Xuzhou Medical University, Jiangsu Xuzhou, China; 2 Jiangsu Center for the Collaboration and Innovation of Cancer, Xuzhou Medical University, Jiangsu Xuzhou, China

## Abstract

Advanced glycation end products/advanced glycation end products receptor (AGEs/AGER) interaction triggers reactive oxygen species (ROS) generation and activates downstream signal pathways and induces apoptosis in endothelial progenitor cells. A number of studies have revealed the involvement of microRNAs (miRNAs) in regulating intracellular ROS production and apoptosis. However, few studies explore the role of miRNAs in regulating the effect of adipose tissue-derived stem cells (ADSCs) in repairing diabetic wound and the associated cellular mechanisms remain unclear. In this study, ADSCs were exposed to AGEs, then siRNA for AGER was transfected into ADSCs. We found that AGEs/AGER axis induced ROS generation and apoptosis in ADSCs. AGEs treatment downregulated miR-5591-5p in ADSCs, which directly targeted AGER. miR-5591-5p suppressed AGEs/AGER axis-mediated ROS generation and apoptosis in ADSCs in vitro. In addition, miR-5591-5p promoted cell survival and enhanced the ability of ADSCs for repairing cutaneous wound in vivo. Furthermore, we confirmed that c-jun kinase (JNK) signal was involved in the inhibitory effect of miR-5591-5p on AGEs/AGER axis-induced ROS generation and apoptosis in ADSCs. Thus, these results indicated that miR-5591-5p targeting AGEs/AGER/JNK signaling axis possibly regulates the effect of ADSCs in repairing diabetic wound.

## Introduction

Adipose tissue-derived stem cells (ADSCs) are derived from adipose tissue stroma, which harbor the ability of self-renewal and differentiate into a number of functional cells^1^. Emerging evidence has shown the beneficial effects of ADSCs administration to treat various diseases because of their simple isolation techniques, easy expandability, low immunogenicity, and pluripotency^2, 3^. Moreover, ADSCs have been found to promote chronic wound healing^4^. Diabetic patients are much more susceptible to developing chronic wounds. ADSCs therapy could potentially influence the treatment of wounds in non-diabetic conditions, but no effect in diabetic patients^5^. Increased apoptosis in stem cells has been considered to impair wound healing in a diabetic rat model^6^. Although efforts has been made to improve cell survival after implantation, more simple and efficient interventions by which to protect ADSCs against apoptosis and increase the therapeutic effect of ADSCs are still required.

Advanced glycation end products (AGEs) refer to a group of heterogeneous macromolecules that are produced by the post-translational modification of proteins via non-enzymatic glycation, lipids and nucleic acids, accumulate with age, and are abundantly elevated in diabetic patients^7^. The increased AGEs in diabetic patients cause a number of pathological changes. There has been evidence that elevated AGEs promotes apoptosis of endothelial progenitor cell (EPC) and endothelial cell inhibits proliferation of repairing cells, thus impedes wound healing^8–10^. Numerous articles have documented that AGEs induce cell apoptosis and may involve in the pathogenesis of biophysical disorders^11, 12^. AGEs provide the bridge between intracellular and extracellular damage through the specific receptor for advanced glycation end products receptor (AGER). AGER is a 45-kDa transmembrane receptor, which belongs to the immunoglobulin superfamily. It is found to be expressed highly during embryonic development, but less expressed in adult tissues^13^. However, pathological conditions of high glucose, reactive oxygen species (ROS), hypoxia, pro-inflammatory mediators, or AGEs itself induce AGER expression^13, 14^. AGE/AGER interactions lead to a diverse array of signaling pathways activation, such as p38 and JNK that participate in apoptosis^15, 16^.

MicroRNAs (miRNAs) are ubiquitously expressed short non-coding RNAs of 20–22 nucleotides, which regulate messenger RNAs (mRNAs) after transcription by targeting the untranslated regions. This leads to degradation of the target mRNAs and/or translation inhibition^17^. Recently, several miRNAs have been demonstrated to interfere with and modulate intracellular apoptosis signaling^18, 19^. However, few studies explore the role of miRNAs in AGEs/AGER signaling related to diabetic wound healing. In this study, we focused on miR-5591-5p via a miRNA array after ADSCs exposure to AGEs. Then, we focused on the role of miR-5591-5p in ADSCs exposed to AGEs, and found that miR-5591-5p regulated the effect of ADSCs in repairing diabetic wound healing via targeting AGES/AGER/JNK signaling axis.

## Results

### AGEs induces AGER expression, ROS generation, and cell apoptosis in ADSCs

**Fig. 1:**
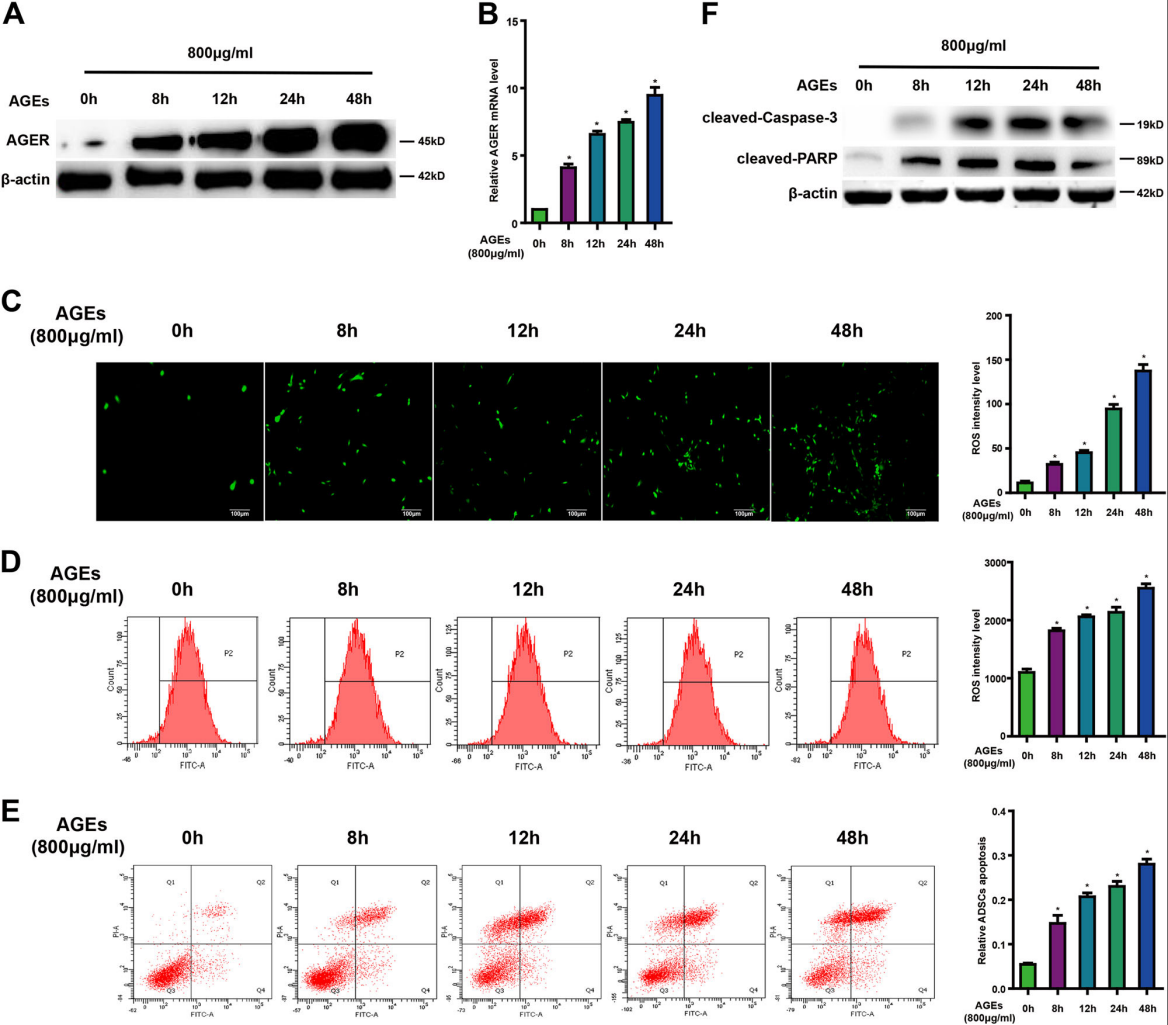
Different concentration of AGEs on expression of AGER, ROS generation, and apoptosis in ADSCs. **a** Representative western blot images showing the protein expression levels of AGER when ADSCs treated with different concentration of AGEs. **b** qPCR analysis of mRNA levels of AGER after cells treated with different concentration of AGEs. **c** Intracellular ROS production was observed under the fluorescence microscope. **d** The level of DCF-sensitive ROS was examined by a flow cytometer. **e** ADSCs were stained with annexin V-FITC/PI and immediately analyzed by flow cytometry. **f** Western blot analysis of protein levels of caspase-3 and PARP. Every experiment repeated at least three times. Error bars indicate mean ± SD (*P < 0.05)

The cellular effects of AGEs are mainly mediated through the receptor for AGEs. To investigate whether AGEs affect the expression of AGER in ADSCs, cells were incubated with or without AGEs (100–1600 μg/ml) for 24 h, the expression of AGER was established by western blot and quantitative PCR. As shown in Figure 1a, b, the upregulation of AGER in response to AGEs was identified in a dose-dependent manner. Fluorescence microscope and flow cytometer showed that production of ROS was increased after AGEs treatment (Fig. 1c, d). In addition, data from flow cytometer displayed that apoptotic cells were increased with AGEs (Fig. 1e). Caspase-3 and PARP are the principal apoptosis markers through which the mitochondrial and cytosolic pathways induce apoptosis. Consequently, we examined the activity of caspase-3 and PARP. Western blot analysis indicated that AGEs treatment promoted caspase-3 and PARP activity (Fig. 1f).

**Fig. 2:**
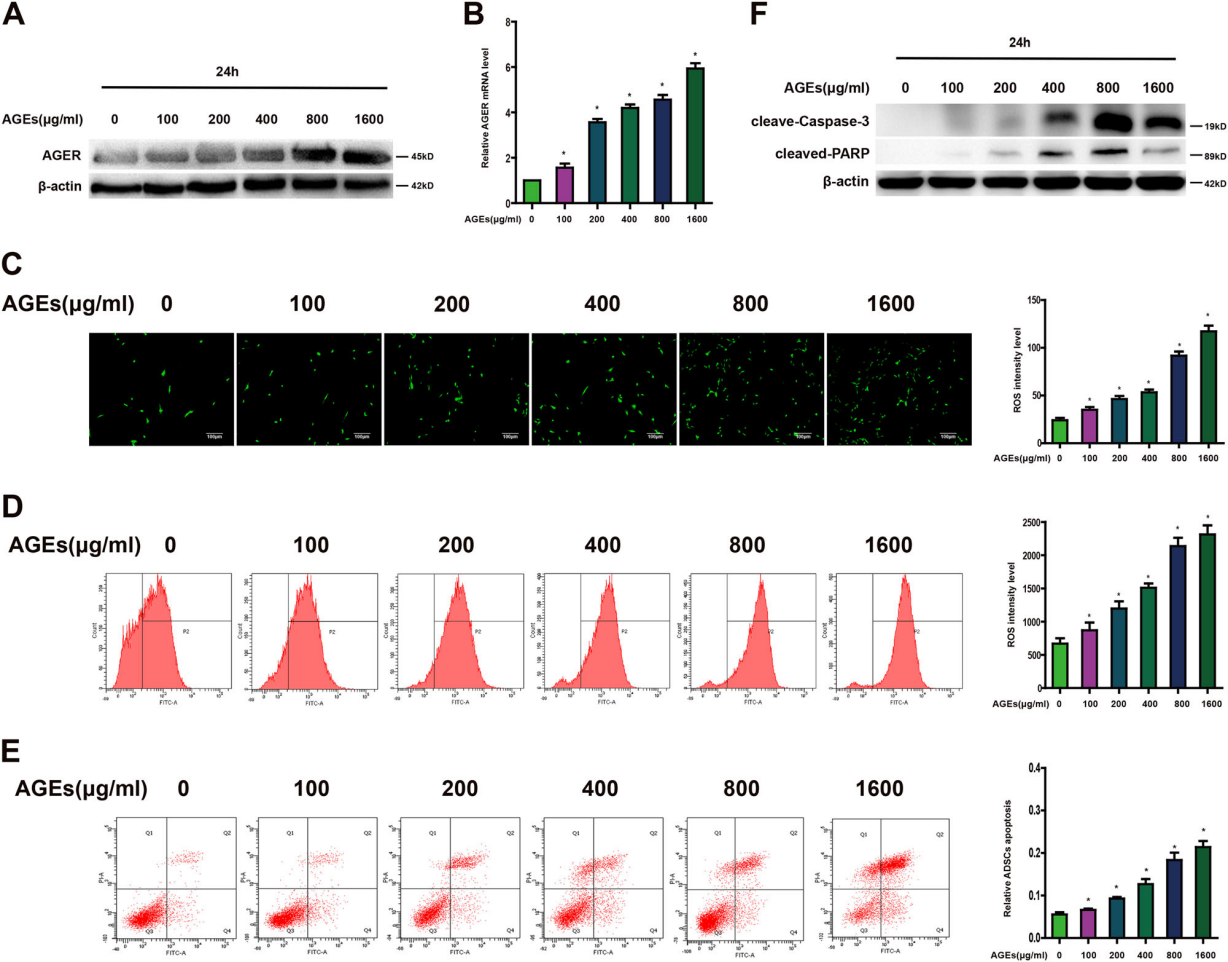
Time course of AGEs on expression of AGER, ROS generation, and apoptosis in ADSCs. **a** Time course of AGER protein expression in ADSCs treated with AGEs (800 μg/ml). **b** qPCR analysis of mRNA levels of AGER after cells treated with AGEs at different time. **c** Intracellular ROS generation was visualized under the fluorescence microscope. **d** Flow cytometer was employed to detect ROS level. **e** ADSCs were stained with annexin V-FITC/PI and immediately analyzed by flow cytometry. **f** Western blot analysis of protein levels of caspase-3 and PARP. Every experiment repeated at least three times. Error bars indicate mean ± SD (*P < 0.05)

Moreover, expression of AGER in ADSCs following treatment with 800 μg/ml AGEs was determined at the different time points. Our results displayed a obvious increase of AGER from 0 to 48 h (Fig. 2a, b). At the same time, ROS level showed distinct elevation from 0 to 48 h in Figure 2c, which is consistent with the time course of enhanced fluorescence probe expression treated with AGEs in Figure 2d. The apoptotic cells were increased in a time course after given AGEs (Fig. 2e). The upregulation of cleaved-caspase-3 and PARP in response to AGEs was also shown in a time-dependent manner (Fig. 2f).

### AGEs/AGER axis induces ROS generation and apoptosis in ADSCs

**Fig. 3:**
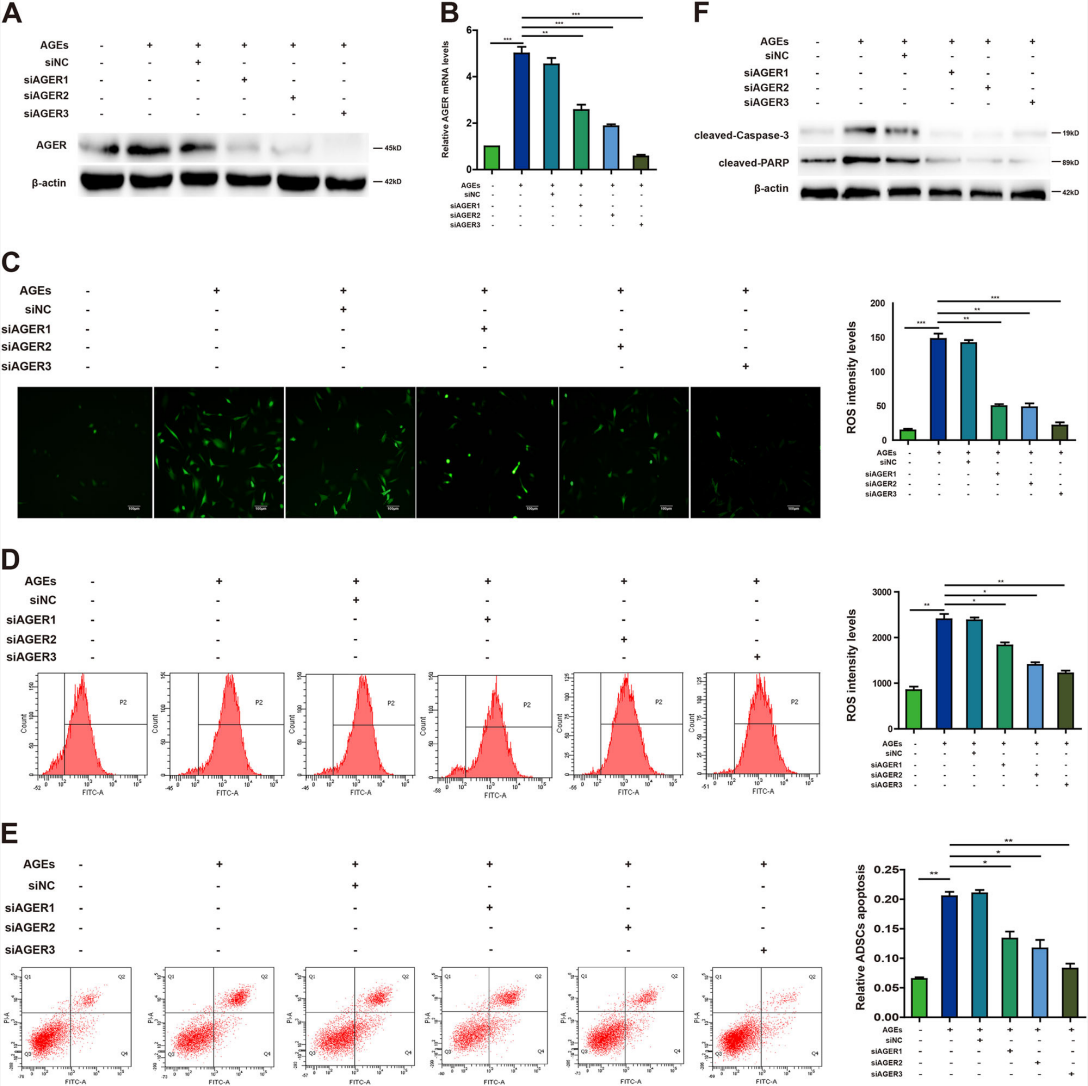
The effect of AGEs/AGER axis on ROS generation and apoptosis in ADSCs. **a** Western blot shows the protein expression of AGER after transfection with siRNAs for AGER in ADSCs. β-actin was used as an internal control. **b** qPCR analysis of mRNA levels of AGER after cells were transfected with siRNAs for AGER. **c** Intracellular ROS generation was observed under the fluorescence microscope. **d** Flow cytometer was employed to detect ROS level. **e** ADSCs were stained with annexin V-FITC/PI and immediately analyzed by flow cytometry. **f** Western blot assays of protein levels of caspase-3 and PARP. Every experiment repeated at least three times. Error bars indicate mean ± SD (*P < 0.05; **P < 0.01; ***P < 0.001)

AGEs can activate diverse signal transduction cascades and downstream pathways, including generation of ROS, leading to apoptosis. To investigate whether AGER is essential for the role of AGEs in ADSCs, we first detected AGER protein and mRNA levels after using small-interfering RNAs (siRNAs) for AGER. The data showed that AGER was enhanced after exposure to AGEs, but declined significantly after transfected with siRNAs for AGER (Fig. 3a, b). Fluorescence microscope showed that production of ROS was increased after AGEs treatment, but diminished after knockdown of AGER, which was consistent with the results of flow cytometer (Fig. 3c, d). Furthermore, we explored whether AGEs/AGER was involved in the regulation of apoptosis in ADSCs. Flow cytometer showed that AGEs induced apoptosis, but this was reversed by inhibition of AGER expression (Fig. 3e). Western blot assay indicated that siRNAs for AGER prevented caspase-3 and PARP from cleavage to suppress caspase signal pathway (Fig. 3f). These data revealed that AGEs/AGER axis induced ROS generation and apoptosis in ADSCs.

### AGER is a direct target of miR-5591-5p

**Fig. 4:**
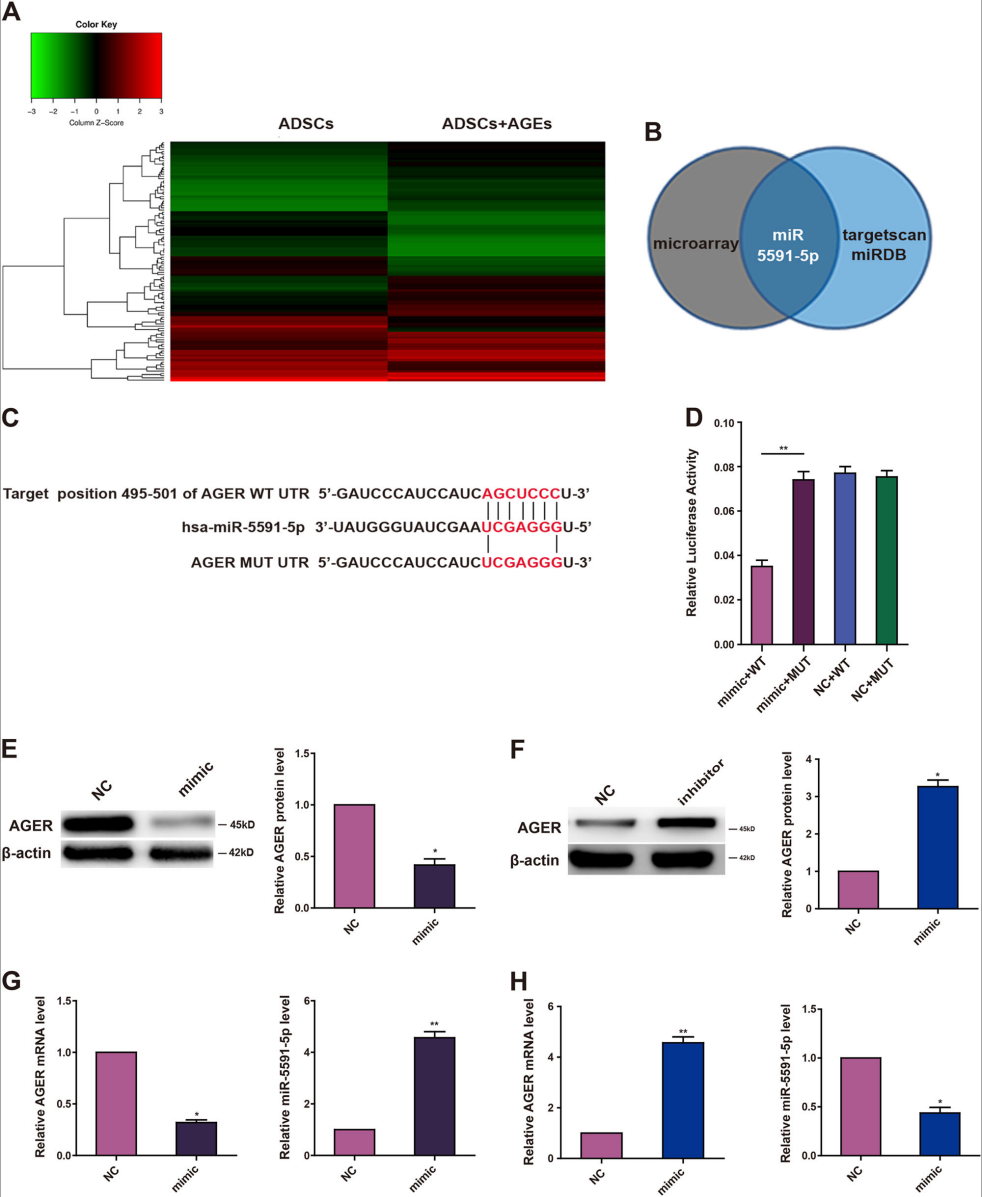
AGER is a target of miR-5591-5p. **a** A heat map showing differentially regulated miRNAs in ADSCs treated with or without AGEs. A color scale is shown below. **b** Schema for identification of the putative miRNA(s) that could be downregulated when cells were given AGEs and partly complementary to a conserved site within the 3′ untranslated region of AGER. **c** The predicted site of miR-5591-5p binding to the AGER 3′-UTR was detected using bioinformatics tools. **d** Mutated site in the AGER 3′-UTR is shown. The effect of miR-5591-5p on luciferase activity induced by the psiCHECK™-2-AGER-WT and psiCHECK™-2-AGER-MUT reporter plasmids in ADSCs. **e**, **f** Western blot images showing the protein expression levels of AGER when ADSCs transfected with miR-5591-5p mimics and inhibitors. **g**, **h** qPCR analysis of mRNA levels of miR-5591-5p and AGER after cells were transfected with miR-5591-5p mimic and inhibitor. Data are shown as means ± SD of three replicates (*P < 0.05; **P < 0.01)

Because miRNAs have been reported to regulate intracellular ROS production and apoptosis signaling in endothelial cells during diabetes^20, 21^, we used a miRNA array to analyze the miRNA profiles in AGEs-treated ADSCs and control cells. The data showed a large number of miRNAs that were downregulated in AGEs-treated ADSCs (Fig. 4a). We then employed miRDB and targetscan softwares to analyze these differentially expressed miRNAs. Only miR-5591-5p was found to be partially complementary to the conserved site in the 3′-untranslated region (UTR) of AGER mRNA (Fig. 4b). To confirm whether miR-5591-5p targets AGER mRNA, we cloned the 3′-UTR sequence of AGER into the psiCHECK™-2 vector. The data showed that introduction of miR-5591-5p diminished luciferase activity of this reporter. In order to verify whether miR-5591-5p specifically inhibited AGER through its potential binding site, we constructed the mutated (mutant type, MUT) reporter at miR-5591-5p-binding site. Our data showed that activity of the MUT 3′-UTR reporter was unchanged (Fig. 4c, d). Moreover, we evaluated the effects of miR-5591-5p on AGER mRNA and protein levels in ADSCs by quantitative PCR (qPCR) and western blot. We found that transfection of miR-5591-5p mimics into ADSCs negatively regulated AGER expression in ADSCs (Fig. 4e, f). Taken together, these findings suggested that miR-5591-5p could directly target the 3′-UTR of AGER and downregulate AGER expression.

### miR-5591-5p suppresses AGEs/AGER axis-mediated ROS generation and apoptosis in ADSCs

**Fig. 5:**
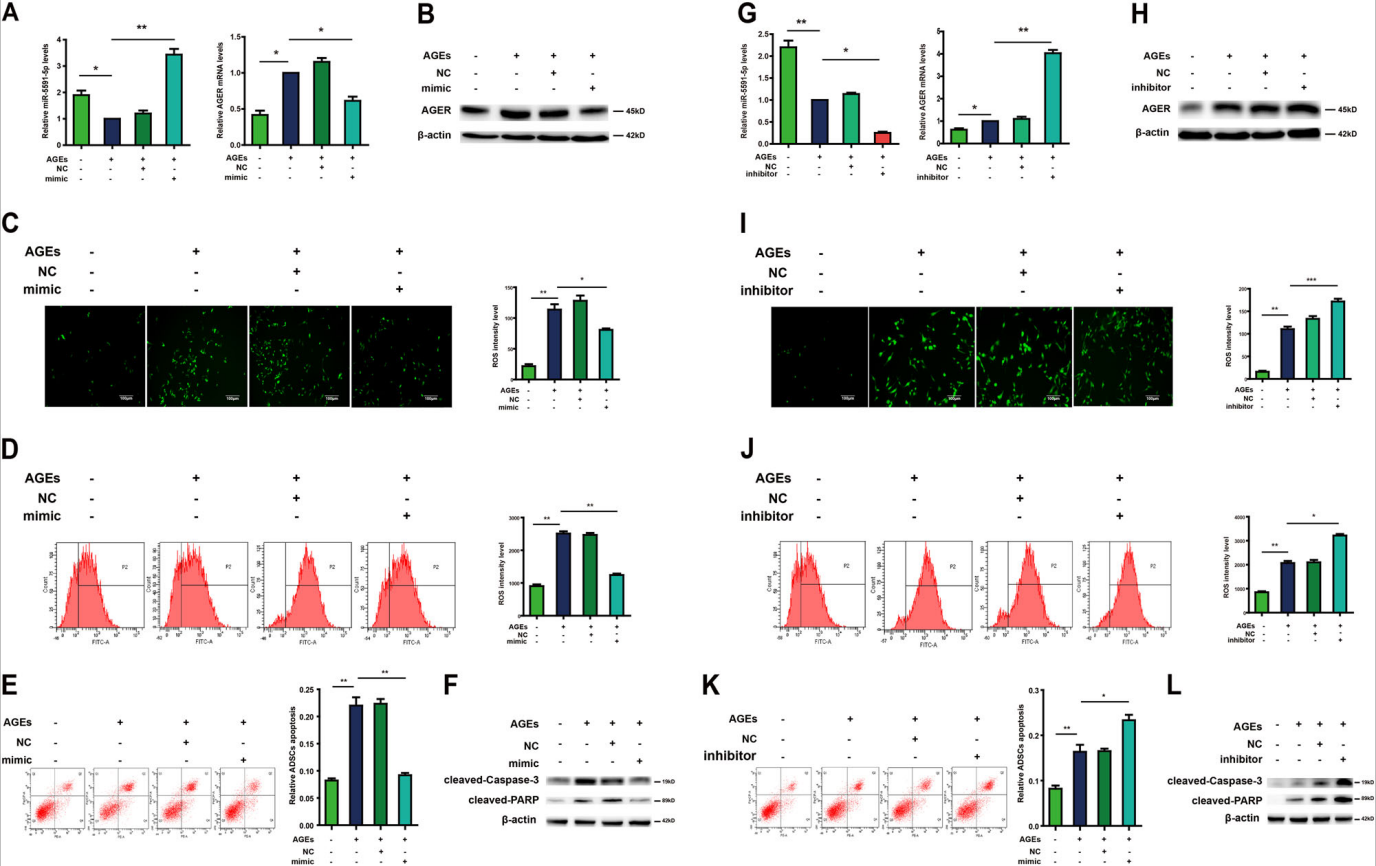
miR-5591-5p suppresses AGEs/AGER axis-mediated ROS generation and apoptosis in ADSCs. **a** qPCR analysis of mRNA levels of miR-5591-5p and AGER after cells were transfected with miR-5591-5p mimics with or without AGEs. **b** Western blot shows the protein expression of AGER after cells were transfected with miR-5591-5p mimics with or without AGEs. **c** Intracellular ROS generation was visualized under the fluorescence microscope after cells were transfected with miR-5591-5p mimics. The scale bars represent 100 μm. **d** The level of DCF-sensitive ROS was measured by a flow cytometer after cells were transfected with miR-5591-5p mimics. **e** Treated ADSCs were stained with annexin V-FITC/PI and immediately analyzed by flow cytometry after cells were transfected with miR-5591-5p mimics. **f** Western blot analysis of protein levels of caspase-3 after cells were transfected with miR-5591-5p mimics with or without AGEs. **g** The mRNA levels of miR-5591-5p and AGER after cells were transfected with miR-5591-5p inhibitors with or without AGEs. **h** Western blot assay of AGER protein levels after cells were transfected with miR-5591-5p inhibitors with or without AGEs. **i** Intracellular ROS generation was observed under the fluorescence microscope after given miR-5591-5p inhibitors. The scale bars represent 100 μm. **j** The level of DCF-sensitive ROS was detected by a flow cytometer after cells were transfected with miR-5591-5p inhibitors. **k** Treated ADSCs were stained with annexin V-FITC/PI and analyzed by flow cytometry after cells were transfected with miR-5591-5p inhibitors. **l** Western blot analysis of protein levels of caspase-3 after cells were transfected with miR-5591-5p inhibitors with or without AGEs. Data are shown as means ± SD of three replicates (*P < 0.05; **P < 0.01; ***P < 0.001)

Because AGER is a target of miR-5591-5p, we further verified whether miR-5591-5p regulated ROS generation and cell apoptosis induced by AGEs/AGER. For the ROS generation, the data showed that miR-5591-5p diminished production of ROS, which was induced by AGEs (Fig. 5a–d). In addition, data of flow cytometer showed that miR-5591-5p also suppressed AGEs-induced apoptosis (Fig. 5e). Western blot analysis for cleaved-caspase-3 and PARP suggested that transfected miR-5591-5p mimics inhibited caspase-3 and PARP activity (Fig. 5f). Conversely, miR-5591-5p inhibitors promoted ROS production induced by AGEs (Fig. 5g–j). Flow cytometer showed that miR-5591-5p inhibitors facilitated AGEs-induced apoptosis (Fig. 5k). The activity of caspase-3 and PARP was elevated by miR-5591-5p inhibitors (Fig. 5l). When we co-transfected vector of AGER and miR-5591-5p mimics in ADSCs, data from supplementary Figure 1 showed that overexpression of AGER promoted ROS generation and apoptosis of ADSCs. However, this positive effect of AGER on ROS generation and apoptosis can be suppressed by miR-5591-5p mimics in ADSCs. These results indicated that miR-5591-5p may act as a negative modulator of AGEs/AGER-induced apoptosis in ADSCs.

### Phosphorylation of JNK signal is involved in AGEs/AGER-induced ROS generation and apoptosis in ADSCs

**Fig. 6:**
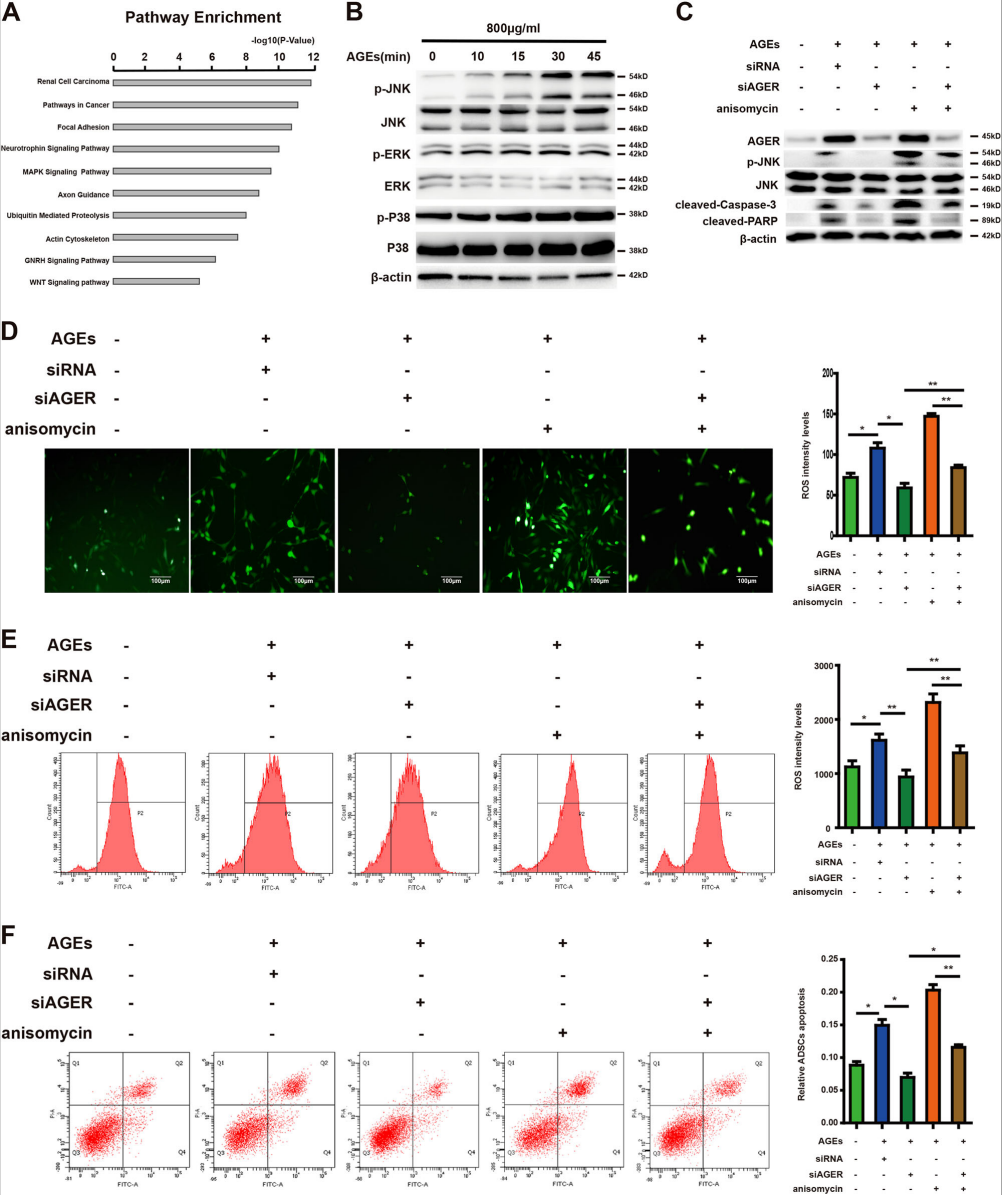
Role of JNK signal in AGEs/AGER-induced ROS generation and apoptosis in ADSCs. **a** Top 10 pathways that are enriched for the AGEs targets. **b** Time course analysis of phosphorylated and total p38, ERK, and JNK protein expression in ADSCs treated with AGEs (800 μg/ml). **c** ADSCs pretreated with AGEs were given AGER siRNA and JNK activator, anisomycin (1 μg/ml). The protein levels of phosphorylated and total JNK, AGER, cleaved-caspase-3 cleaved-PARP, and β-actin were determined by western blot analysis. **d** Intracellular ROS generation was visualized under the fluorescence microscope. **e** Flow cytometer was employed to detect ROS level. **f** Treated ADSCs were stained with annexin V-FITC/PI and immediately analyzed by flow cytometry. Each value is expressed as the mean ± SD of three independent experiments (*P < 0.05; **P < 0.01; ***P < 0.001)

To investigate the potential downstream pathway that regulated by AGEs/AGER, we performed KEGG pathway analysis to search for the potential signal pathway that regulated by AGES. The top 10 KEGG pathways enriched for the AGEs targets were displayed in Figure 6a. Previous studies have reported that the MAPK pathway is critical for production of ROS and induction of apoptosis, we asked if AGEs/AGER axis triggers the MAPK pathway in ADSCs. We first detected the phosphorylation of MAPK family members (ERK1/2, JNK, and p38 MAPK) after treated with AGEs. As shown in Figure 6b, exposure to AGEs significantly increased JNK activation in a time-dependent manner, but no change of ERK1/2 and p38 phosphorylation.

To further determine whether JNK pathway is activated by AGEs/AGER axis, ADSCs transfected with siRNA for AGER were treated with JNK activator, anisomycin (800 μg/ml). As shown in Figure 6c, both AGEs and anisomycin treatment induced JNK phosphorylation, resulted in high expression of cleaved-caspase-3 and PARP, but this phosphorylation could be blocked by siRNA for AGER. In addition, knockdown AGER also inhibited caspase-3 pathway, blocked AGEs or anisomycin-induced upregulation of ROS generation and apoptosis of ADSCs (Fig. 6d–f). Moreover, we employed JNK inhibitor SP600125 to confirm the role of JNK activity in this process. Data from supplementary Figure 2 showed that SP600125 significantly inhibited ROS generation and apoptosis, which was induced by AGEs. So, these results suggested that JNK pathway was required in AGEs/AGER-mediated ROS generation and cell apoptosis.

### JNK signal is involved in the inhibitory effect of miR-5591-5p on AGEs/AGER-induced ROS generation and apoptosis in ADSCs

**Fig. 7:**
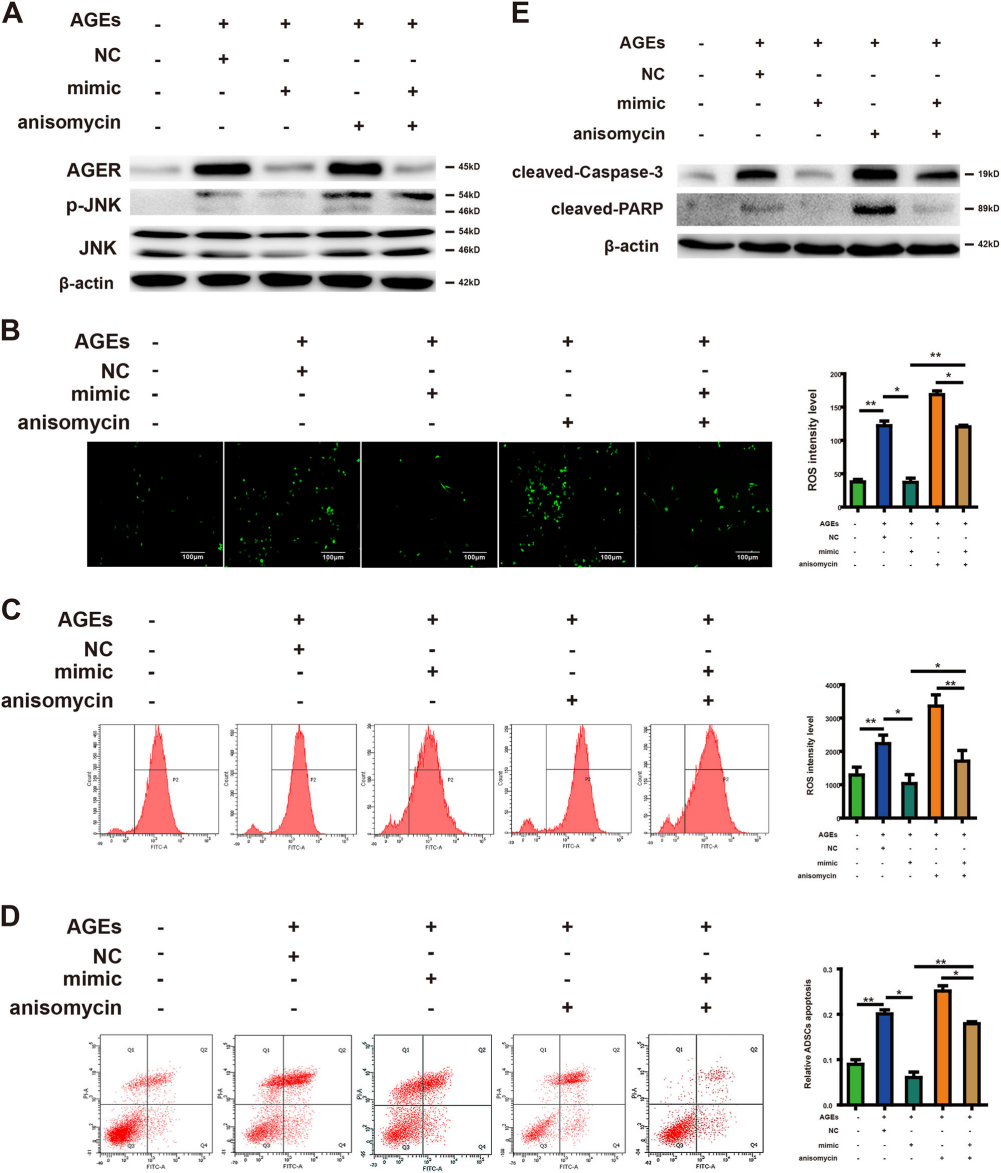
Role of JNK signal in the inhibitory effect of miR-5591-5p in ADSCs. **a** ADSCs pretreated with AGEs were given AGER siRNA and miR-5591-5p mimics. The whole-cell lysates were analyzed for the protein levels of p-JNK, JNK, AGER, and β-actin were determined by using western blot analysis. **b** Intracellular ROS generation was observed under the fluorescence microscope. **c** Flow cytometer was employed to detect ROS level. **d** Treated ADSCs were stained with annexin V-FITC/PI and immediately analyzed by flow cytometry. **e** Western blot analysis of protein levels of cleaved-caspase-3 and PARP after cells pretreated with AGEs were given AGER siRNA and miR-5591-5p mimics. β-actin was used as an internal control. Each value is expressed as the mean ± SD of three independent experiments (*P < 0.05; **P < 0.01)

Since JNK signal is responsible for the regulation of ROS generation and apoptosis mediated by AGEs/AGER, we wanted to identify whether miR-5591-5p could influence the activity of JNK induced by AGEs/AGER in ADSCs. From Fig. 7a, we can see that although AGEs or anisomycin treatment resulted in phosphorylation of JNK, this was overturned by miR-5591-5p mimics. Moreover, miR-5591-5p mimics suppressed the positive effect of AGEs or anisomycin on ROS generation (Fig. 7b, c). The apoptotic ADSCs were also decreased by miR-5591-5p mimics through activated caspase pathway (Fig. 7d, e). So, we think that miR-5591-5p may suppress JNK signal activity, which lead to ROS generation and apoptosis in ADSCs.

### miR-5591-5p promotes cell survival and cutaneous wound healing in vivo

**Fig. 8:**
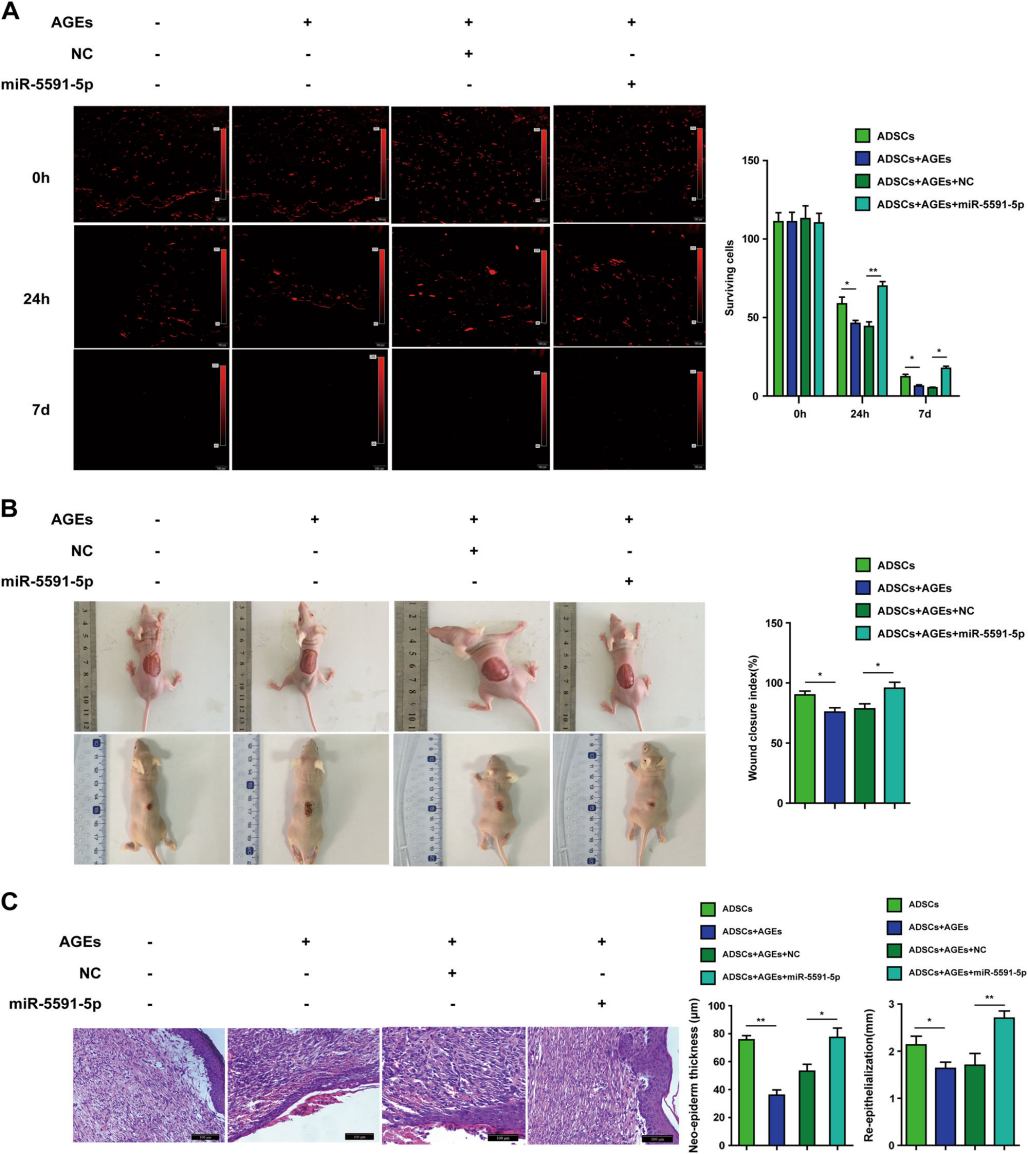
miR-5591-5p promotes cell survival and cutaneous wound healing of diabetic mice. **a** Fluorescence microscope was used to observe CM-Dil fluorescence after CM-Dil marked ADSCs, which were transfected with miR-5591-5p mimics with or without AGEs, were injected into chronic wounds of diabetic mice in 0 h, 24 h, and 7 days, respectively. **b** Gross appearance of the skin wounds 7 days after given ADSCs, which were transfected with miR-5591-5p mimics with or without AGEs. **c** Representative image of H&E staining after injection of cells transfected with miR-5591-5p mimics with or without AGEs (*P < 0.05; **P < 0.01)

To determine the effect of miR-5591-5p on cutaneous wound healing in vivo, we first used CM-Dil marked ADSCs to inject into the chronic wounds of diabetic mice. Only a few cells pretreated with AGEs survived in the wound bed. However, more cells expressing miR-5591-5p could be found surviving in the wound bed (Fig. 8a).

**Fig. 9:**
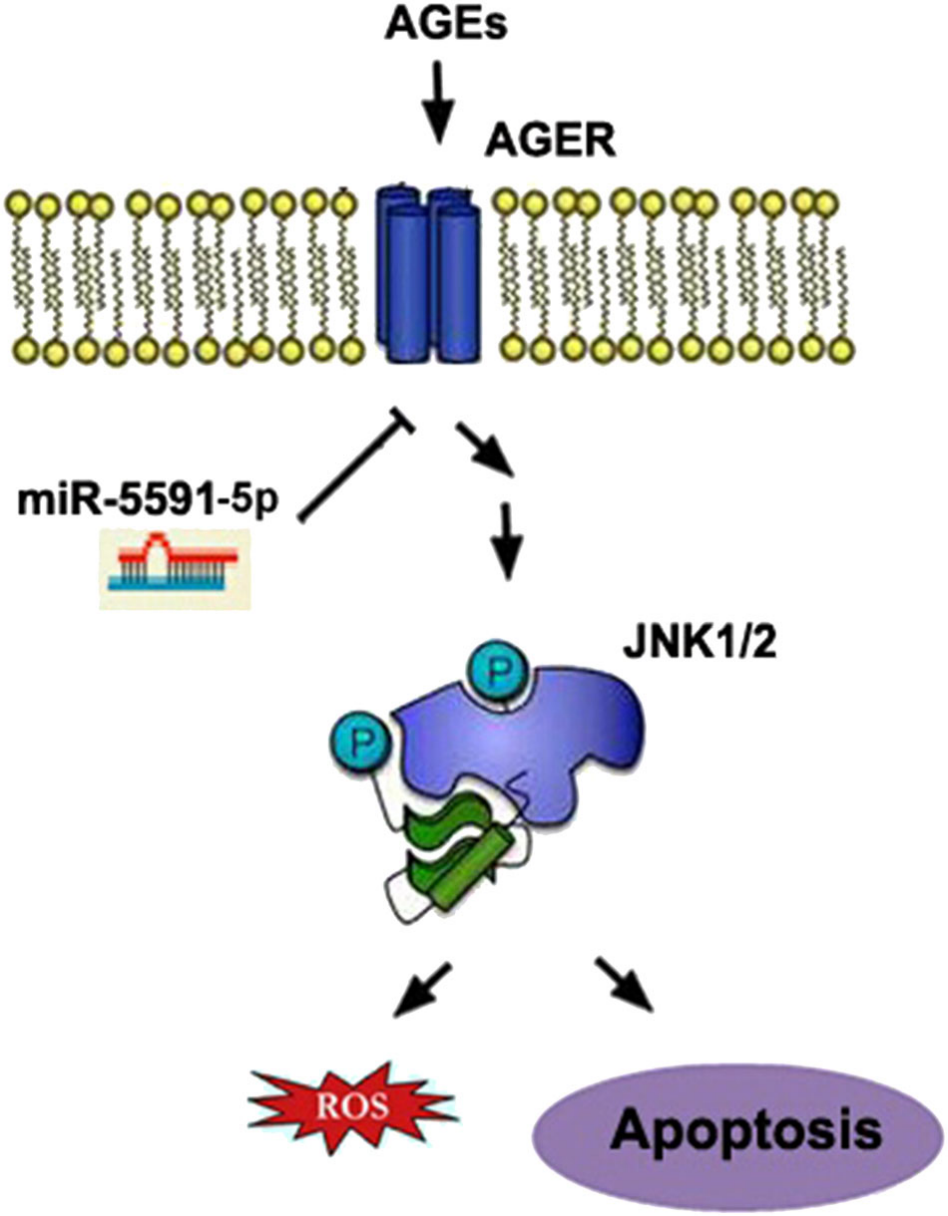
Schematic diagram of miR-5591-5p regulates ROS generation and apoptosis via AGEs/AGER/JNK signaling axis. We speculate that miR-5591-5p can suppress ROS generation and apoptosis owing to the inhibition of AGEs/AGER/JNK pathway by direct target AGER

We further explore whether forced-expression of miR-5591-5p in ADSCs can be prone to the wound healing in diabetic mice. As displayed in Figure 8b, the wound area of diabetic mice was significantly greater when ADSCs pretreated with AGEs. However, ADSCs with miR-5591-5p showed smaller wound area when compared with ADSCs or AGEs pretreated ADSCs, the wound closure index was significantly better than that in AGEs groups (Fig. 9).

In addition, hematoxylin and eosin (H&E) staining indicated that AGEs-pretreating significantly impair the repairing ability of ADSCs. However, ADSCs with miR-5591-5p showed greater repairing ability by promoting epithelization of the wound. The thickness of neo-epiderm and re-epithelization in the miR-5591-5p group were significantly greater than that in the control group (0.8 °C).

## Discussion

Although recent studies have shown beneficial effects of ADSCs administration for various diseases, impairment of resident and recruited cell functions strongly results in delays in wound healing under diabetic conditions. Extensive research has showed the vital role of diabetes in regulating stem cell apoptosis in the pathogenesis of delayed wound healing^22, 23^. However, the mechanism of diabetes in the damage of ADSCs functions in repairing wound healing has been not elucidated.

AGEs are noxious metabolic products that accumulate in diabetic patients but not in normal individuals. Excessive formation of AGEs is considered as the most important mechanism that triggers the pathophysiological cascades associated with the onset of diabetic complications. There has been evidence that the elevated AGEs in diabetic patients bring pathological damages, such as promotion of EPC and endothelial cell apoptosis, thus impedes wound healing^8–10^. AGER is widely viewed as the best-characterized AGEs receptor, which is responsible for most of the negative effects of AGEs. Extensive research has suggested that intracellular AGE signaling is mediated via AGER present in most cell types^13, 14^. Apoptosis is a potential mechanism through which AGEs exert its effects on cellular dysfunction^24^. Excessive production of ROS plays a critical role in the process of apoptosis^25^. Previous studies indicate that AGEs/AGER interaction triggers ROS generation and activates downstream JNK pathways and induce apoptosis in EPCs^26^. AGEs/AGER signaling have been demonstrated to induce ROS production and apoptosis via the JNK and p38 MAPK pathways in osteoblasts and fibroblasts^15, 27^. However, few studies about the AGEs/AGER signaling in ADSCs has been reported. One recent study has demonstrated that AGEs induce apoptosis of ADSCs in vitro via AGER-dependent p38 MAPK pathway^28^. Furthermore, they find that AGE-induced apoptosis is suppressed by antioxidants N-acetylcysteine (NAC) and ascorbic acid 2-phosphate (AAP)^29^. In this study, we demonstrated that the incubation of ADSCs with AGEs resulted in significant upregulation of AGER and cell apoptosis. The interaction of AGEs with AGER regulated increased production of ROS and leads to apoptosis through caspase-3 activity.

miRNAs are a group of small, non-coding, and single-strand RNAs that control gene expression at the post-transcriptional and translational level by matching their seed sequences to complementary sequences in the 3′-UTR of target mRNAs^17^. Previous studies revealed dysregulated miRNA expression under AGEs^30^. However, AGEs-mediated alteration of miRNA expression in ADSCs has not been well characterized. Using an miRNA microarray, we surveyed the differential expression of miRNAs after AGEs treatment in ADSCs. The results showed that a panel of miRNAs in AGE-treated ADSCs were significantly dysregulated, which may regulate the ROS production and apoptosis of ADSCs under AGEs. Diabetic conditions can regulate miRNA activity. It has been reported that high glucose reduced EZH2 binding to the miR-101 locus, whereas EZH2 overexpression inhibits miR-101 promoter activity in human fetal endothelial cells of the umbilical cord vein^31^. Diabetic conditions induce miR-379 cluster expression, depends on the expression of the host long non-coding RNA transcript from its promoter^32^. Consequently, as metabolic products in diabetic, AGEs likely control miRNAs expression through regulating their promoter activity.

A number of studies have revealed the involvement of miRNAs in regulating intracellular ROS production and apoptosis. miR-210 is considered to induce apoptosis in colorectal cancer cells via a ROS-dependent mechanism^33^. Loss of miR-137 is confirmed to ameliorate high-glucose-induced damage in human umbilical vein endothelial cells (HUVECs) by overexpression of AMPKα1, leading to decreasing oxidative stress^34^. However, only a few studies investigate the role of miRNAs in regulating AGE/AGER signaling related to diabetic manifestations and during diabetes. A genome-wide study reveals elevation of miR-214 after treating primary monocytes and monocytic cell line (THP-1) with various AGEs. This is also observed in patients with chronic renal failure^30^. In addition, antioxidants NAC and AAP upregulate miR-223 in ADSCs. miR-223 mimics blocked antioxidant inhibition of AGE-induced apoptosis and ROS generation^29^. In the miRNA microarray, we selected miR-5591-5p because it was the only one that was predicted to target AGER. We demonstrated that miR-5591-5p could directly bind to the 3′-UTR of AGER. miR-5591-5p regulated ROS generation and cell apoptosis induced by AGEs/AGER in ADSCs. Furthermore, miR-5591-5p promoted ADSCs survival and improved the repairing ability of ADSCs for cutaneous wound healing of diabetic mice. These findings indicated the important role of miR-5591-5p in regulating the effect of ADSCs in repairing diabetic wound healing via targeting AGEs/AGER.

MAPK signals are frequently over-activated in a variety of disease states. The activated MAPK pathways have been implicated in mediating apoptotic responses. AGEs have been reported to stimulate the activation of MAPK cascades in different cell types^16, 35, 36^. It has been shown that AGEs induces apoptosis of ADSCs in vitro via AGER-dependent p38 MAPK pathway^28^. More and more miRNAs have been identified that modulate MAPK pathway in stem cells. Mei et al.^37^ find that miR-21 promotes adipogenesis and osteogenesis of MSCs through modulating the ERK-MAPK signaling pathway. In addition, miR-126 is considered to promote mesenchymal stem cells differentiation toward endothelial cells via activating PI3K/Akt and MAPK/ERK pathway^38^. To further investigate whether MAPK signals are involved in regulating the effect of ADSCs on repairing diabetic wound healing by miR-5591-5p, we first detected the levels of phosphorylated forms of MAPK family members after treated with AGEs. We found that JNK signal pathway involved in the regulation of ROS generation and apoptosis mediated by AGEs/AGER. Then, we examined whether miR-5591-5p can influence the activity of JNK induced by AGEs/AGER in ADSCs. Our results revealed that miR-5591-5p may suppress JNK signal activity, which causes ROS generation and apoptosis of ADSCs.

In summary, we have shown that the interaction of AGEs with AGER regulated increased production of ROS and leads to apoptosis of ADSCs. miR5591-5p can regulate the effect of ADSCs in repairing diabetic wound healing via targeting AGES/AGER axis. Moreover, JNK signal is involved in the inhibitory effect of miR-5591-5p on AGEs/AGER-induced ROS generation and apoptosis in ADSCs. Our studies uncover the mechanism of miR-5591-5p that regulate specifically the effect of ADSCs in repairing diabetic wound via targeting AGES/AGER/JNK signaling in order to formulate improved and optimized miRNA-targeted approaches for therapy of diabetic wound healing.

## Materials and methods

### Isolation and culture of human ADSCs

All the methods were carried out as described in our previous study^4^. Adipose tissue samples were obtained from three liposuction aspirates of patients with informed consent at the Affiliated Hospital of Xuzhou Medical University. Isolated ADSCs were maintained in l-Dulbecco’s modified Eagle’s medium (DMEM; Invitrogen, CA, USA) containing 10% fetal bovine serum (FBS; Invitrogen) in 37 °C humidifed incubator with 5% CO_2_.

### Cell treatment

AGEs were obtained from Sigma-Aldrich (St. Louis, MO, USA). AGEs were added to ADSCs and then subject to some experiments. Also, a JNK activator, anisomycin (0.1 μg/ml, Beyotime, Shanghai, China) was used to activate the JNK signaling pathway. A JNK inhibitor, SP600125 (20 μg/ml, Beyotime, Shanghai, China) was added to cultured cells to block the JNK activity in this study.

### Cell transfection

The AGER siRNAs were purchased from GenePharma (Shanghai, China). The sequences for the AGER siRNA used for the experiments were as follows:

AGER siRNA1: 5′-GCCAACCCAGAAGCTAGAA -3′;

AGER siRNA2: 5′- GTGAATCCTGCCTCTGAAC-3′;

AGER siRNA3: 5′- GCCTCTGAACTCACAGCCA-3′.

ADSCs were plated in six-well or 96-well plates and transient transfection using Lipofectamine2000 (Invitrogen) according to the manufacturer’s instructions.

Stable transfection for cells was performed as previously described^39^. LV5-Control and LV5-miR-5591-5p lentiviral vectors were purchased from Shanghai GenePharma Company (China). Lentiviruses mixed with polybrene (5 mg/ml) were added to ADSCs and puromycin (5 mg/ml) was applied to select positive clones. Stable miR-5591-5p transfectants were isolated after 2 weeks.

### RNA extraction and qPCR

Total RNA was extracted and complementary DNA was generated as previously described^40^. qPCR was employed on a 7500HT Fast qPCR instrument (New York, USA) as previously described^40^. The primer sets were shown in Table 1.

**Table 1:** Primer sequences

Name	Primer
AGER	Sense: 5′-GGCTGGTGTTCCCAATAAGG-3′; anti-sense: 3′-TCACAGGTCAGGGTTACGGTTC-5′
miR-5591-5p	Sense: 5′-GATGCCCATGCCGATTCTT-3′; anti-sense: 3′-ACTGGGCTTCGATTTCACCT-5′
miR-5591-5p inhibitor	Sense: 5′-GATGCCCATGCCGATTCTT-3′; anti-sense: 3′-ACTGGGCTTCGATTTCACCT-5′

### Measurement of intracellular ROS

Cells were seeded in a six-well plate plated with 5 × 10^4^ cells per well. Cells were added with 10 μM fluorescent probe CM-H2DCFDA (Molecular Probes; Invitrogen), incubated for 60 min at 37 °C. After washing with PBS, cells were detected using fluorescence microscope (Olympus, Tokyo, Japan) and FACSCalibur flow cytometer (BD Biosciences, Franklin Lakes, NJ, USA).

### Apoptosis assay

Following treatment, ADSCs were stained with fluorescein (FITC)-conjugated annexin V and propidium iodide (FITC/PI) (KeyGen Biotech, Nanjing, China), and analyzed on flow cytometer to determine rate of apoptosis.

### Luciferase reporter gene assay

ADSCs were transfected with either psiCHECK-2/AGER 3′-UTR, miR-5591-5p mimics or control mimics, and then subjected to luciferase assay using the dual-luciferase reporter assay kit (Promega, Madison, WI, USA) according to instructions.

### Western blot

Cell extracts were separated on SDS-polyacrylamide gel, then proteins were transferred to a nitrocellulose membrane and incubated with the following antibodies: rabbit polyclonal against AGER (1:500; Santa Cruz, CA, USA), cleave-caspase3 (1:800), cleave-PARP (1:800), JNK (1:800), p-JNK (1:500), ERK (1:800), and p-ERK (1:1000), p38 (1:500), p-p38 (1:500), and rabbit monoclonal against β-actin (1:1000; Cell Signaling Technology, Danvers, MA, USA). Immunoreactive protein bands were detected with Tanon scanning system.

### Diabetic wound model

Six-week-old nu/nu athymic nude mice were purchased from Vital River (Beijing, China) and maintained under specific pathogen-free conditions. All animal studies were approved by Animal Care and Use Committee. Diabetic mice were induced by intraperitoneal injection of 150 mg/kg STZ (Sigma, Shanghai, China). Blood glucose level in tail venous blood samples was measured 14 days later. Animals with blood glucose levels above 300 mg/dl and apparent features of polydipsia, polyphagia, and polyuria were considered to be diabetic. After anesthetizing animals with intraperitoneal injections of chloral hydrate (10%, 5 ml/kg), a full thickness skin defect of 2-cm diameter was made on the back. The wound area was analyzed as previously described^4^.

### ADSCs survival assay in vivo

ADSCs were marked with CM-Dil prior to injection. When ADSCs after the third passage reached 70–80% confluency, they were incubated with 5 μg/ml CM-Dil at 37 °C for 30 min and then washed with PBS three times. The efficiency of the CM-Dil staining was detected with a fluorescence microscope. Following treatment, 1 × 10^6^ ADSCs were injected around the wound bed. At 0 h, 24 h, and 7 days after transplantation, under anesthesia the wounds were collected with the surrounding tissue. The skin tissues were embedded in OCT compound (Tissue-Tek^®^, Miles Laboratories, Naperille, IL, USA) at −80 °C. CM-Dil-labeled ADSCs were observed to examine CM-Dil fluorescence as whole mounts under a fluorescence microscope with excitation at 420 nm and emission at 480 nm (Olympus, Tokyo, Japan).

### H&E staining

Seven days after transplantation, the wounds with the surrounding tissue were collected and fixed in 10% formalin, and H&E staining was performed to observe the epithelization^41^. Images were collected by a Camedia Master C-3040 digital camera (Olympus).

### Statistical analysis

Results were presented as mean ± SD and statistically analyzed utilizing a Student’s *t*-test with SPSS software (SPSS 16.0, Inc., Chicago, IL, USA). *P* < 0.05 was considered as a statistically significant difference.

## Electronic supplementary material

Supplementary Figure Legends

Supplementary Figure 1

Supplementary Figure 2
