# Supplementary material for: miR-5591-5p regulates the effect of ADSCs in repairing diabetic wound via targeting AGEs/AGER/JNK signaling axis
Source: Cell Death Dis. 2018 May 11;9(5):566. doi: 10.1038/s41419-018-0615-9 (PMC5948214; doi:10.1038/s41419-018-0615-9)
Supplement: Supplementary file 1 — Supplementary Figure Legends [file 41419_2018_615_MOESM1_ESM.docx]

**Supplementary Figure 1. Effect of introduction of AGER on miR-5591-5p regulates ROS generation and apoptosis in ADSCs.** **A.** ADSCs pretreated with AGEs (800 μg/ml), and then co-transfected vector of AGER and miR-5591-5p mimics. The whole cell lysates were analyzed for the protein levels of AGER by Western blot. **B.** Intracellular ROS generation was observed under the fluorescence microscope. **C.** Flow cytometer was employed to detect ROS level. **D.** Treated ADSCs were stained with Annexin V-FITC/PI and immediately analyzed by flow cytometry. **E.** Western blot analysis of protein levels of cleaved-caspase-3 and PARP after cells pretreated with AGEs were transfected with AGER vector and miR-5591-5p mimics. β-actin was used as an internal control. Each value is expressed as the mean ± SD of three independent experiments (**P*<0.05; ***P*<0.01; ****P*<0.001).

**Supplementary Figure 2. Effect of JNK inhibitor SP600125 on ROS generation and apoptosis induced by AGEs/AGER in ADSCs. A.** ADSCs pretreated with AGEs were given JNK inhibitor, SP600125 (20 μg/ml). The whole cell lysates were analyzed for the protein levels of AGER and β-actin by Western blot. **B.** Intracellular ROS generation was observed under the fluorescence microscope. **C.** Flow cytometer was employed to detect ROS level. **D.** Treated ADSCs were stained with Annexin V-FITC/PI and immediately analyzed by flow cytometry. **E.** Western blot analysis of protein levels of cleaved-caspase-3 and PARP after cells pretreated with AGEs were given SP600125. β-actin was used as an internal control (***P*<0.01; ****P*<0.001).
