# Supplementary figures and images for: miR-5591-5p regulates the effect of ADSCs in repairing diabetic wound via targeting AGEs/AGER/JNK signaling axis
Source: Cell Death Dis. 2018 May 11;9(5):566. doi: 10.1038/s41419-018-0615-9 (PMC5948214; doi:10.1038/s41419-018-0615-9)

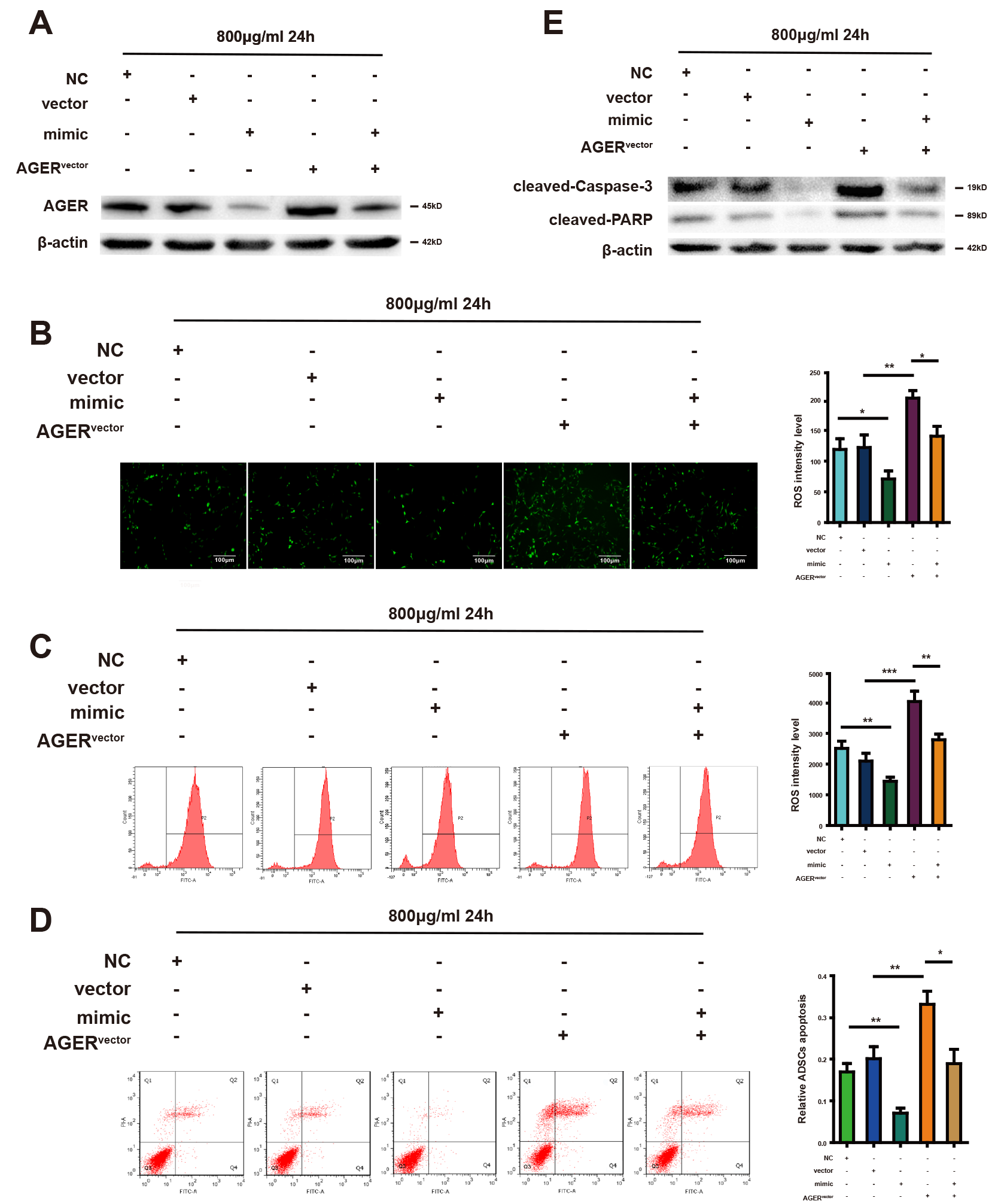

Supplement: Supplementary file 2 — Supplementary Figure 1 [file 41419_2018_615_MOESM2_ESM.tif]

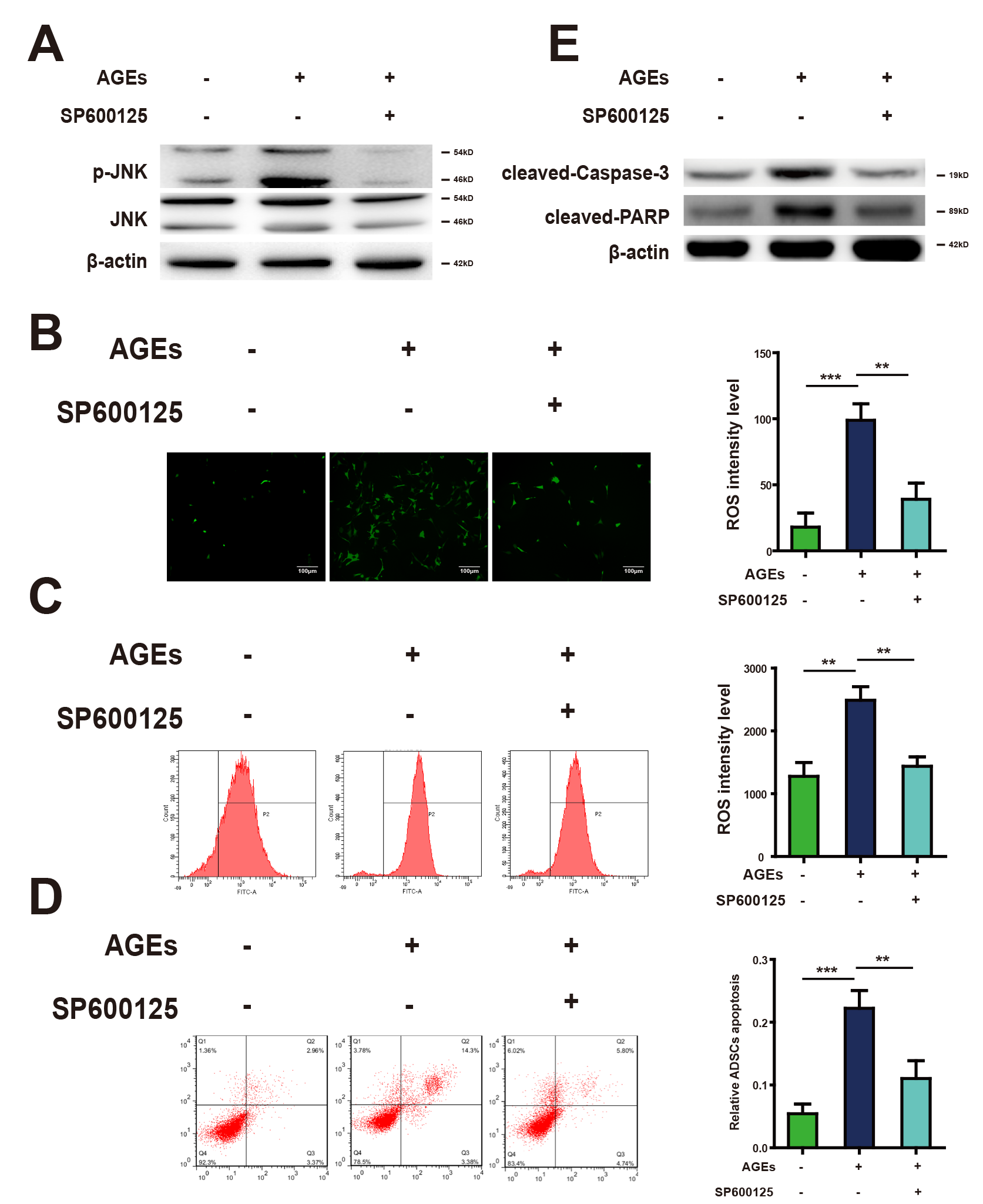

Supplement: Supplementary file 3 — Supplementary Figure 2 [file 41419_2018_615_MOESM3_ESM.tif]
